# Noncommunicable diseases risk factors and the risk of COVID-19 among university employees in Indonesia

**DOI:** 10.1371/journal.pone.0263146

**Published:** 2022-06-06

**Authors:** Indah Suci Widyahening, Dhanasari Vidiawati, Trevino A. Pakasi, Pradana Soewondo, Abdillah Ahsan

**Affiliations:** 1 Department of Community Medicine, Faculty of Medicine, Universitas Indonesia, Jakarta Pusat, Indonesia; 2 Southeast Asian Ministers of Education Organization—Regional Centre for Food and Nutrition (SEAMEO-RECFON)—Pusat Kajian Gizi Regional (PKGR), Universitas Indonesia, Jakarta, Indonesia; 3 Department of Internal Medicine, Faculty of Medicine, Universitas Indonesia–Cipto Mangunkusumo Hospital, Jakarta Pusat, Indonesia; 4 Department of Economics, Faculty of Economics and Business, Universitas Indonesia, Depok, Indonesia; The University of Mississippi Medical Center, UNITED STATES

## Abstract

**Introduction:**

Noncommunicable diseases (NCDs) are still a major public health problem in Indonesia. Studies have shown that risk factors of NCDs are associated with coronavirus disease 2019 (COVID-19) severity and mortality. However, it is unclear whether NCD risk factors are also risks for new COVID-19 cases. This study aimed to obtain an NCD risk profile among university employees and its associations with contracting COVID-19.

**Methods:**

A cross-sectional study was conducted in October 2021. Participants were administrative employees of Universitas Indonesia (UI), Depok City, West Java. Assessment of NCD risk factors was based on the World Health Organization STEPwise approach to NCD risk factor surveillance (WHO STEPS). Demographic, working, and medical-history data were obtained electronically by using a Google Form. Physical and laboratory examinations were done in the Integrated Post for NCDs. Risks were expressed as adjusted odds ratio (OR_adj_) and 95% confidence interval (CI) in multivariate analyses.

**Results:**

A total of 613 employees were enrolled. Men were predominant (54.8%), and about 36% of them work in shift as security personnel. About 66.7% were overweight or obese and 77.8% had hypertension. There were 138 (22.8%) employees who had COVID-19. Nearly all (95.6%) had been fully vaccinated against COVID-19. At-risk waist circumference (OR_adj_ 1.72, 95% CI 1.15–2.56, *p* = 0.008) and total cholesterol level of 200–239 mg/dL (OR_adj_ 2.30, 95% CI 1.19–4.44, p = 0.013) were independent risk factors, but shift work (OR_adj_ 0.52, 95% CI 0.34–0.80, *p* = 0.003) was protective against COVID-19.

**Conclusion:**

The prevalence of NCD risk factors among university administrative employees was high, increasing the risk of contracting COVID-19. A behavioral intervention program to manage NCD risk factors at the university level is urgently needed according to the Health Promoting University framework.

## Introduction

The coronavirus disease 2019 (COVID-19) pandemic caused by severe acute respiratory syndrome coronavirus 2 (SARS-CoV-2) is ongoing and has possibly become endemic. Currently, community transmission of COVID-19 in Indonesia has remained at a low level after a second dramatic peak in mid-July 2021 [1]. However, the actual number of cases may be higher than officially registered; the weighted estimate of seroprevalence of the SARS-CoV-2 antibody in Jakarta in March 2021 was 44.5%. This implies that almost half of the population has been infected with SARS-CoV-2 [2].

Noncommunicable diseases (NCDs) are major health problems in the world. The national Basic Health Research in 2018 has shown increasing prevalence of NCDs in Indonesia, such as diabetes (from 6.9% in 2013 to 10.9% in 2018) and hypertension (from 25.8% in 2013 to 34.1% in 2018) [3]. Early in the COVID-19 pandemic, patients needing intensive care were more likely to have NCDs as comorbidities (hypertension, diabetes, cardiovascular disease, and cerebrovascular disease) [4].

The association between NCD risk factors and COVID-19 is not clear. Previous data showed that body mass index (BMI) and obesity are associated with SARS-CoV-2 infection, hospitalization, and mortality [5, 6]. Obesity seems to play an important role in the pathogenesis of COVID-19 [7]. Multimorbidity, especially renal, cardiovascular, and metabolic morbidities, is associated with a higher risk of a COVID-19 positive test [8, 9].

To prevent new infections, we need to identify modifiable risk factors against contracting COVID-19, such as obesity and/or components of metabolic syndrome. This is important because factors that can be modified to reduce risk will be the target for health education along with a rigorous vaccination program. This study aimed to obtain the NCD risk profiles among university employees and its association with contracting COVID-19.

## Methods

### Study design and participants

The design of this study was cross-sectional and conducted in October 2021. Participants were employees of the University Central Administration, Universitas Indonesia (UI) and the administrative/supporting staffs of the Faculty Medicine of the university. Demographic, working, and medical-history data were obtained electronically by using a Google Form, which was distributed through the WhatsApp group of the Directorate of Human Resources and the Faculty of Medicine. Participants were then invited to attend physical and laboratory examination in the Integrated Post for NCDs throughout Universitas Indonesia at the main campus in Depok City, West Java and the Salemba campus in Jakarta (total six posts). This post was a community-based program oriented toward promotive and preventive efforts to control NCDs [10].

Sample size was calculated using the formula for a prevalence study with a confidence level of 0.05 and precision of 5%. Hence, a minimum number of 384 subjects was expected. Participants were enrolled in this study if they provided written consent. Ethics approval was granted from the Health Research Ethic Committee of the Faculty of Medicine Universitas Indonesia–Cipto Mangunkusumo Hospital (No. KET-1006/UN2.F1/ETIK/PPM.00.02/2021). Written informed consent was requested from all participants prior to the study.

### History of COVID-19 and vaccination

A diagnosis of COVID-19 was established as a history of a positive PCR or rapid-detection test for SARS-CoV-2 from nasopharyngeal swabs. However, since this type of question could be seen as a sensitive question to some of the participants, we offered a “prefer not to answer” option as an answer choice. A history of COVID-19 was counted once between March 2020 and October 2021; re-infection, if it had occurred, was not considered. Participants were also asked whether or not they were hospitalized as a result of having COVID-19.

The COVID-19 vaccination campaign began in January 2021 (in the UI Hospital), and by mid-2021, most non-medical university employees had been scheduled for two doses.

### Assessment of noncommunicable diseases risk factors

Assessment was based on the World Health Organization STEPwise approach to NCD risk factor surveillance (STEPS) [11]. The survey instrument includes: tobacco use, alcohol use, physical inactivity, unhealthy diet, and key biological risk factors: overweightness and obesity, raised blood pressure, raised blood glucose, and abnormal blood lipids. Risk factors assessed in this study consisted of anthropometric measurements (BMI and waist circumference), medical history (hypertension, diabetes, dyslipidemia), working pattern (shift or non-shift), behavioral risk factors (smoking; alcohol consumption; fruit, vegetable, and salt consumption; level of physical activity), and blood chemistry results (fasting blood glucose; triglyceride; and total, low-density lipoprotein (LDL), and high-density lipoprotein (HDL) cholesterol levels).

Body weight was measured using a weight scale (SECA, Hamburg, Germany), and body height was measured using a stature meter microtoise (GEA medical). BMI was calculated as body weight in kilograms divided by body height in meters squared. Nutritional status was then classified based on the BMI criteria for an Asian population as follows: underweight (BMI <18.5 kg/m^2^), normal weight (18.5–22.9 kg/m^2^), overweight (23.0–24.9 kg/m^2^), obesity class I (25.0–29.9 kg/m^2^), and obesity class II (>30.0 kg/m^2^) [12]. Waist circumference (WC) was measured using a measuring tape (Onemed Waist Ruler OD 235). At-risk WC was >102 cm for men and >88 cm for women.

Systolic and diastolic blood pressures (BP) were measured using a digital blood pressure monitor (Beurer® BM 58, Germany). Similar measurement tools were used for all participants. Hypertension Elevated blood pressure was indicated if systolic BP was ≥130 mmHg and/or diastolic BP ≥85 mmHg. A minimum of 8 h fasting was required for the measurement of fasting blood glucose (FBG), triglyceride (TG), and cholesterol levels. The result was indicated as high if FBG ≥110 mg/dL, TG ≥ 150 mg/dL, total cholesterol ≥200 mg/dL, and LDL-cholesterol ≥100 mg/dL; while HDL-cholesterol was considered low if ≤40 mg/dL, based on the metabolic syndrome criteria by the American Heart Association/National Heart, Lung, and Blood Institute [13]. Physical activity was considered low (inactive) if less than 150 min of moderate-intensity physical activity or less than 75 min of vigorous-intensity physical activity per week was reported [14].

### Statistical analyses

Baseline characteristics of the participants and risk factors were presented descriptively. Univariate logistic regression test was performed for each risk factor. Multivariate logistic regression analyses were done to estimate the adjusted odds ratio (OR_adj_) and its corresponding 95% confidence intervals (CIs) for the association between risk factor and SARS-CoV-2 infection. Adjustments were made to all other variables included in the multivariate logistic regression (variables that had a *p* value of <0.2 in the univariate analysis). Missing data were excluded from the analysis including the “prefer not to answer” response. All statistical analyses were done using Statistical Package for Social Science (SPSS) version 20. A *p* value of less than 0.05 was considered significant.

## Results

### Characteristics of the study subjects

From 789 supporting staff members invited, 750 attended the examination, 618 completed the questionnaires, and 5 did not undergo laboratory tests. In total, 613 (77.7%) participants were included in the final analyses. However, there were missing data in several variables (less than 2% participants for each variable). Table 1 shows the characteristics and health history of the university employees. Male participants were predominant (54.8%). Around 76.7% of them were 40 years old or younger, and 72.4% were married. About 36% (222 of 613) work in shifts as security personnel. There were 138 (22.8%) employees who had had COVID-19, but only 14 (2.3%) had required hospitalization. Nearly all the participants (94.0%) were fully vaccinated against COVID-19.

**Table 1:** Characteristics and health history of the university employees (*n* = 613).

Variables	n (%)
Age*; mean (SD): 34.81 (15.10)	
≤ 40 years	470 (77.7)
> 40 years	135 (22.3)
Sex	
Male	336 (54.8)
Female	277 (45.2)
Marriage status	
Married	444 (72.4)
Un-married	169 (27.6)
Working in shift	
Yes	222 (36.2)
No	391 (63.8)
History of hypertension*	
Yes	143 (23.6)
No	462 (76.4)
History of diabetes*	
Yes	21 (3.5)
No	584 (96.5)
History of dyslipidemia*	
Yes	103 (17.1)
No	501 (82.9)
History of COVID-19*	
Yes	138 (22.8)
No	467 (77.2)
History of COVID-19 hospitalization*	
Yes	14 (2.3)
No	592 (97.7)
COVID-19 vaccination*	
Complete	576 (95.5)
In-complete	17 (2.8)
None	10 (1.7)

*Variable with missing data (less than 2% of participants for each variable)

Table 2 shows the distribution of behavioral risk factors for NCDs among the university employees. The proportion of those who currently smoke is nearly 25% (151 of 613) and 15.2% (93 of 613) admitted consuming alcohol. The proportion of those who consume fruit 5–7 days per week was just below 20% (119 of 613), while the percentage of those who consume vegetables was also low (below 50%) (282 of 613). The majority of the respondents (94.3%) were categorized as having an active physical activity level.

**Table 2:** Behavioral risk factors for non-communicable diseases among university employees (*n* = 613).

Variable	*n* (%)
Current smoking	
Yes	151 (24.6)
No	462 (75.4)
Alcohol consumption	
Yes	93 (15.2)
No	520 (84.8)
Fruit consumption*	
Never	12 (2.0)
1–4 days/week	479 (78.5)
5–7 days/week	119 (19.5)
Vegetable consumption*	
Never	5 (0.8)
1–4 days/week	324 (53.0)
5–7 days/week	282 (46.2)
Salt consumption*	
Far too much or too much	113 (18.5)
Just the right amount	312 (51.1)
Too little or far too little	112 (18.3)
Don’t know	74 (12.1)
Physical activity level	
Inactive	35 (5.7)
Active	578 (94.3)

*Variable with missing data (less than 2% of participants for each variable)

Table 3 shows biological risk factors for NCDs among the university employees. Two-thirds of them (409 of 613) were categorized as overweight and obese, more than 75% (477 of 613) had increased blood pressure, half (320 of 613) had an at at-risk waist circumference, and almost half (294 of 613) had increased fasting blood sugar levels.

**Table 3:** Biological risk factors for noncommunicable diseases among university employees (*n* = 613).

Variable	n (%)
BMI (kg/m^2^)	
Underweight (<18.5)	32 (5.2)
Normoweight (18.5–22.9)	172 (28.1)
Overweight (23–24.9)	106 (17.3)
Obese I (25–29.9)	200 (32.6)
Obese II (>30)	103 (16.8)
Waist circumference (cm)	
Normal (male ≤90, female ≤80)	293 (47.8)
At risk (male >90, female >80)	320 (52.2)
Systolic blood pressure (mmHg)	
<120	195 (31.8)
120–139	286 (46.7)
≥140	132 (21.5)
Diastolic blood pressure (mmHg)	
<80	221 (36.1)
80–89	234 (38.2)
≥90	158 (25.8)
Blood pressure (mmHg)	
systolic <120 and diastolic <80	136 (22.2)
systolic 120–139 OR diastolic 80–89	281 (45.8)
systolic ≥140 OR diastolic ≥90 OR both	196 (32.0)
Fasting blood glucose (mg/dL)	
<100	319 (52.0)
100–125	244 (39.8)
≥126	50 (8.2)
Total cholesterol level (mg/dL)	
<200	561 (91.5)
200–239	42 (6.9)
≥240	10 (1.6)
LDL-cholesterol level (mg/dL)	
<100	488 (79.6)
100–129	102 (16.6)
≥130	23 (3.8)
HDL-cholesterol level (mg/dL)	
>40	484 (79.0)
≤40	129 (21.0)
Triglyceride level (mg/dL)	
<150	569 (92.8)
150–199	32 (5.1)
≥200	12 (2.0)

Univariate analyses between each risk factors with SARS-CoV-2 infection are presented in the (S1–S3 Tables). Table 4 shows the risk factors associated with SARS-CoV-2 infection based on multivariate logistic regression. The final model of multivariate logistic regression revealed that shift work (adjusted OR [OR_adj_] 0.52, 95% confidence interval [CI] 0.34–0.80, p = 0.003), having an at-risk waist circumference (OR_adj_ 1.72, 95% CI 1.15–2.56, p = 0.008), and a total cholesterol level of 200–239 mg/dl (OR_adj_ 2.30, 95% CI 1.19–4.44, p = 0.013) are variables that have a significant relationship with a history of COVID-19.

**Table 4:** Risk factors associated with COVID-19 among university employees (*n* = 605).

Variables	OR_adj_*	95%CI	*p*
Shift work			
No	Ref		
Yes	0.52	0.34–0.80	**0.003**
Waist circumference (cm)			
Normal (male ≤90, female ≤80)	Ref		
At risk (male >90, female >80)	1.72	1.15–2.56	**0.008**
Total cholesterol (mg/dl)			
<200	Ref		
200–239	2.30	1.19–4.44	**0.013**
≥240	2.80	0.72–10.87	0.137

*Variables included in the multivariate logistic regression (*p*-value of <0.2 in the univariate analysis) were shift work, smoking, BMI, waist circumference, systolic blood pressure, and total cholesterol.

## Discussion

Our study showed that more than half of the university employees have at least one biological risk factor associated with NCDs (overweight or obese, increased blood pressure, at-risk waist circumference, and increased fasting blood sugar). Having an at-risk waist circumference and a total cholesterol level of 200–239 mg/dL increased the risk of contracting COVID-19 while working in shift prevented it.

Assessing the NCD risk factors was our university’s commitment to protecting health and promoting the well-being of our university members as part of the Health Promoting University framework [15]. It seems that NCD risk factors are common among university employees. A study in Saudi Arabia also found that more than half of university employees there had three or more NCD risk factors, i.e., 64% were overweight or obese, 22.1% had hypertension, and 21.5% had diabetes [16]. Another study showed that 72% of the university employees and their families were overweight or obese [17]. In Nigeria, the most common risk factors among university employees were inadequate intake of fruit and vegetables (94.6%), physical inactivity (77.8%), and dyslipidemia (51.8%) [18].

Early in the COVID-19 pandemic, obesity was identified as a significant risk factor for severe disease [19, 20]. A population-based cohort study found that excess weight is an important modifiable risk factor for severe COVID-19 outcomes [21]. Meta-analyses confirmed that obesity is a risk factor for developing severe COVID-19 through several possible mechanisms [22]. Obesity is considered a low-grade, persistent inflammation. Pro-inflammatory cytokines are elevated in obesity due to dysregulation of normal adipose homeostasis. Hypertrophic adipocytes lead to insulin resistance and inflammation [23]. These changes might ultimately result in impairment of host immune defense that had been associated with increased susceptibility to severe disease and poor outcomes in obese COVID-19 patients [24]. Little is known about obesity as a risk factor for contracting COVID-19. A recent review suggested that obesity increases susceptibility to SARS-CoV-2 infection due to changes in immune pathophysiology, including the increased production of pro-inflammatory cytokines, adipokines, and leptin [25].

A recent study found that the ratio of adiponectin to leptin (Adpn/Lep) was positively correlated with C-reactive protein level, a marker of systemic inflammation. Increased production of Adpn/Lep was caused by increased adiponectin and reduced leptin; it might be a compensatory response to systemic inflammation [26]. Leptin is an adipocytokine that is involved in various physiological functions, and it maintains homeostasis in the immune system [27]. Obesity, increased leptin, and leptin resistance are associated with severe COVID-19. Leptin is the link between metabolic and immunity responses involving T-cell activation upon an acute respiratory viral infection, including SARS-CoV-2. In obesity, there is a large dysregulation of endocrine and inflammatory events leading to an inadequate immune response to acute SARS-CoV-2 infection [28, 29].

A large cohort study from the general population in the UK found that modifiable risk factors for contracting COVID-19 were a higher BMI, higher glycated hemoglobin, smoking, a slow walking pace (a proxy for physical fitness), and the use of blood pressure medication (a proxy for hypertension). A high level of HDL cholesterol was associated with lower risk. The authors concluded that lifestyle modification might reduce the risk of contracting COVID-19 [30]. Another study found that higher total cholesterol and ApoB levels might increase the risk of SARS-CoV-2 infection [31].

Many studies use BMI as an indicator of obesity, which delineates excessive body fat. However, a higher BMI may not represent a higher amount of body fat since it cannot distinguish between fat and lean body mass [32]. We found that high BMI did not have a significant association with the incidence of COVID-19 but high waist circumference (WC) did; the significance was not lost after adjustment for BMI.

Clinical studies on abdominal obesity and COVID-19 are still evolving. One study found that abdominal obesity was associated with a high chest x-ray severity score better than BMI [33]. A UK study found that high WC was associated with a positive test for the SARS-CoV-2 virus only in people ≥ 65 years, independent of BMI [34]. Another study found a positive association between WC and COVID-19 susceptibility (OR = 1.38; 95% CI: 1.07–1.78; *p* = 0.015). However, the significance was lost after adjustment for BMI. The authors concluded that overall obesity has a causal impact on the susceptibility to COVID-19 and obese people are regarded as high-risk [35].

The ACE2 receptor plays an important role in SARS-COV-2 infectivity as the virus uses the receptor to attach itself to the cell surface [36]. ACE2 is widely expressed in fat and may be the reason why obese patients experience more severe COVID-19 symptoms [37]. A recent study found that the expression of ACE2 in fat tissue (both visceral and subcutaneous) is higher than in lung tissue [38]. Upon binding to the cell surface, SARS-CoV-2 may enter the cell through clathrin-mediated endocytosis of the cell membrane [39]. Human cell membrane contains cholesterol, a key structural lipid that is often used by pathogens for their pathogenesis [40]. Therefore, higher membrane cholesterol provides higher efficiency in viral entry. However, COVID-19 patients have generally shown reduced total cholesterol, HDL, and LDL-cholesterol levels which are associated with disease severity [41]. A systematic review confirmed that lower total, HDL- and LDL-cholesterol levels were significantly associated with COVID-19 severity and mortality but not triglyceride level [42].

In our study, working in shift was a protective factor against contracting COVID-19. These were the security guards who work individually and mostly outdoor, thereby reducing the risk of COVID-19 transmission. On the contrary, a study in the UK found that shift work was associated with increased risk of contracting COVID-19 [43]. More detailed analyses showed that shift work was associated with a higher likelihood of contracting COVID-19 for both irregular (OR 2.42; 95% CI 1.92–3.05) and permanent shift work (OR 2.50; 95% CI 1.95–3.19) [44]. However, unlike our study, the UK cohort enrolled people with various occupations.

There were several limitations in this study. Firstly, the design was not prospective and included only people who survived COVID-19. Therefore, this study potentially suffered from selection and recall biases. Information on the history of risk factors might have also suffered from recall bias, but in the analyses, we used objective data from the current physical examination and laboratory test results. Secondly, we cannot differentiate between whether the participants had COVID-19 before or after vaccination, and most of the employees completed their vaccination schedule about three months before our data collection. Therefore, the effect of the COVID-19 vaccine on the incidence was not known. Universitas Indonesia is one of the most prominent universities in Indonesia, located in an area where the prevalence of NCDs and COVID-19 is the highest in the country. Hence, the prevalence of COVID-19 and NCD risk factors reported in this study might also be higher than other university employees in Indonesia.

## Conclusion

University administrative employees have a substantially high prevalence of NCD risk factors, and this has increased their risk of contracting COVID-19. A behavioral intervention program to manage the NCD risk factors at the university level is urgently needed according to the Health Promoting University framework.

## Supporting information

S1 Table Univariate analysis between characteristics and health history of the university employees with COVID-19 (n = 605). (DOCX)

S2 Table Univariate analysis between behavioral risk factors for non-communicable diseases and COVID-19 among university employees (n = 605). (DOCX)

S3 Table Univariate analysis between biological risk factors for noncommunicable diseases and COVID-19 among university employees (n = 605). (DOCX)
