# Supplementary material for: Noncommunicable diseases risk factors and the risk of COVID-19 among university employees in Indonesia
Source: PLoS One. 2022 Jun 6;17(6):e0263146. doi: 10.1371/journal.pone.0263146 (PMC9170090; doi:10.1371/journal.pone.0263146)
Supplement: S1 Table — (DOCX) [file pone.0263146.s001.docx]

S1 Table. Univariate analysis between characteristics and health history of the university employees with COVID-19 (*n* = 605)

| **Variables** | **History of COVID-19** | |  | **P** |
| --- | --- | --- | --- | --- |
|  | **YES** | **NO** | **OR (95%CI)** |  |
| Age*  < 40 years  > 40 years | 106  29 | 367  92 | 1.09 (0.68-1.75) | 0.689 |
| Sex  Male  Female | 69  69 | 262  205 | 1.28 (0.87-1.87) | 0.206 |
| Marriage status  Married  Un-married | 101  37 | 337  130 | 0.95 (0.62-1.46) | 0.813 |
| Working in shift  Yes  No | 34  104 | 185  282 | 0.50 (0.32-0.77) | **0.001** |
| History of hypertension*  Yes  No | 29  109 | 110  350 | 0.85 (0.53-1.34) | 0.480 |
| History of diabetes*  Yes  No | 4  132 | 17  444 | 0.79 (0.26-2.39) | 0.678 |
| History of dyslipidemia*  Yes  No | 30  108 | 71  387 | 1.51 (0.94-2.44) | **0.087** |

*Variable with missing data (less than 2% of participants for each variable)
