# Supplementary material for: Noncommunicable diseases risk factors and the risk of COVID-19 among university employees in Indonesia
Source: PLoS One. 2022 Jun 6;17(6):e0263146. doi: 10.1371/journal.pone.0263146 (PMC9170090; doi:10.1371/journal.pone.0263146)
Supplement: S2 Table — (DOCX) [file pone.0263146.s002.docx]

S2 Table. Univariate analysis between behavioral risk factors for non-communicable diseases and COVID-19 among university employees (*n* = 605)

| **Variable** | **History of COVID-19** | |  | ***p*** |
| --- | --- | --- | --- | --- |
|  | **YES** | **NO** | **OR (95%CI)** |  |
| Current smoking  Yes  No | 22  116 | 127  340 | 0.51 (0.31-0.84) | **0.007** |
| Alcohol consumption  Yes  No | 18  120 | 73  394 | 0.81 (0.47-1.41) | 0.455 |
| Fruit consumption*  Never  1-4 days/week  5-7 days/week | 1  102  34 | 11  371  83 | 0.22 (0.03-1.79)  0.67 (0.43-1.06) | 0.157  0.086 |
| Vegetable consumption*  Never  1-4 days/week  5-7 days/week | 1  65  72 | 4  254  207 | 0.72 (0.08-6.54)  0.74 (0.50-1.08) | 0.769  0.116 |
| Salt consumption*  Far too much or too much and Don’t know  Just the right amount and  Too little or far too little | 32  106 | 151  314 | 0.63 (0.40-0.98) | **0.037** |
| Physical activity level  Inactive  Active | 27  111 | 91  376 | 0.69 (0.32-1.49) | 0.984 |

*Variable with missing data (less than 2% of participants for each variable)
