# Supplementary material for: Noncommunicable diseases risk factors and the risk of COVID-19 among university employees in Indonesia
Source: PLoS One. 2022 Jun 6;17(6):e0263146. doi: 10.1371/journal.pone.0263146 (PMC9170090; doi:10.1371/journal.pone.0263146)
Supplement: S3 Table — (DOCX) [file pone.0263146.s003.docx]

S3 Table. Univariate analysis between biological risk factors for noncommunicable diseases and COVID-19 among university employees (*n* = 605)

| **Variable** | **History of COVID-19** | |  | **p** |
| --- | --- | --- | --- | --- |
|  | **YES** | **NO** | **OR (95%CI)** |  |
| BMI (kg/m^2^)  <18.5 – <23  23-24.9  >25 | 36  22  80 | 164  84  219 | Ref  1.19 (0.66-2.16)  1.66 (2.07-2.59) | 0.559  **0.024** |
| Waist circumference (cm)  Normal (male <90, female <80)  At risk (male >90, female >80) | 50  88 | 237  230 | Ref  1.81 (1.23-2.68) | **0.003** |
| Systolic blood pressure (mmHg)  <120  120-139  >140 | 37  62  39 | 157  218  92 | Ref  1.21 (0.76-1.90)  1.80 (1.07-3.02) | 0.419  **0.026** |
| Diastolic blood pressure (mmHg)  <80  80-89  >90 | 46  54  38 | 175  175  117 | Ref  1.17 (0.75-1.83)  1.24 (0.76-2.01) | 0.481  0.397 |
| Blood pressure (mmHg)  systolic <120 and diastolic <80  systolic 120-139 OR diastolic 80-89  systolic >140 OR diastolic >90 OR both | 28  62  48 | 108  214  145 | Ref  1.12 (0.68-1.85)  1.28 (0.75-2.17) | 0.665  0.365 |
| Fasting blood glucose (mg/dL)  <100  100-125  >126 | 77  51  10 | 240  188  39 | Ref  0.85 (0.57-1.26)  0.80 (0.38-1.68) | 0.413  0.553 |
| Total cholesterol level (mg/dL)  <200  200-239  >240 | 117  17  4 | 437  25  5 | Ref  2.54 (1.33-4.86)  2.99 (0.79-11.30) | **0.005**  0.107 |
| LDL-cholesterol level (mg/dL)  <100  100-129  >130 | 104  24  10 | 380  75  12 | Ref  1.17 (0.70-1.94)  3.04 (1.28-7.24) | 0.547  **0.012** |
| HDL-cholesterol level (mg/dL)  >40  <40 | 110  28 | 368  99 | Ref  0.95 (0.59-1.52) | 0.818 |
| Triglyceride level (mg/dL)  <150  150-199  >200 | 128  8  2 | 434  24  9 | Ref  1.13 (0.50-2.58)  0.75 (0.16-3.53) | 0.771  0.719 |
